# Key Beliefs for Targeted Interventions to Increase Physical Activity in Children: Analyzing Data from an Extended Version of the Theory of Planned Behaviour

**DOI:** 10.1155/2010/893854

**Published:** 2010-06-21

**Authors:** A. Bélanger-Gravel, G. Godin

**Affiliations:** ^1^Department of Social and Preventive Medicine, Faculty of Medicine, Vandry Pavilion, Laval University, QC, Canada G1V 0A6; ^2^Canada Research Chair on Behaviour and Health, Faculty of Nursing, Vandry Pavilion, Laval University, QC, Canada G1V 0A6

## Abstract

Given the high prevalence of overweight and low levels of physical activity among children, a better understanding of physical activity behaviour is an important step in intervention planning. This study, based on the theory of planned behaviour, was conducted among 313 fifth graders and their parents. Children completed a computer-based questionnaire to evaluate theoretical constructs and behaviour. Additional information was obtained from parents by means of a questionnaire. Correlates of children's physical activity were intention and self-identity. Determinants of intention were self-efficacy, self-identity, and attitude. Parental variables were mediated through cognitions. Among girls, practicing sedentary activities was an additional negative determinant of intention. Key beliefs of boys and girls were related to time management and difficulties associated with physical activity. For girls, social identification as an active girl was another important belief related to positive intention. This study provides theory-based information for the development of more effective interventions aimed at promoting physical activity among children.

## 1. Introduction

In North America, the prevalence of overweight and obese children has increased considerably in recent decades, affecting about one third of youths [1–4]. Although regular physical activity is a healthy way to control body weight, few children are physically active [5–7]. This phenomenon is even more prevalent during adolescence [8, 9], when a major decrease in physical activity levels is frequently observed [6]. Consequently, paying more attention to youths' physical activity behaviour before they give up physical activity could help to prevent this withdrawal. 

Results from previous reviews of correlates of physical activity among children revealed inconsistencies in the most important factors related to this behaviour [10, 11]. Moreover, most studies reviewed were not based on sound theoretical frameworks such as the theory of planned behaviour (TPB) [12] or the social cognitive theory (SCT) [13]. Consequently, although a number of interventions have been developed and implemented, results from a recent review of such studies showed that most of the interventions have experienced limited success [14]. According to some authors, the effectiveness of physical activity interventions would benefit from a better understanding of this behaviour [15–17]. Thus, analyzing data from a well-recognized theoretical framework for the identification of intervention targets might prove to be an interesting way to increase physical activity more effectively among children [18–20].

**Figure 1:**
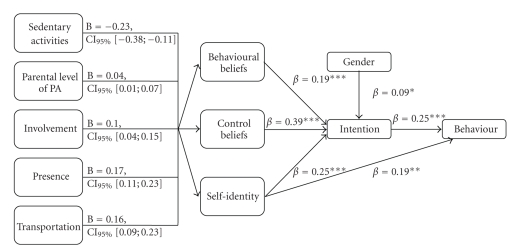
Final model explaining children's physical activity behaviour and intention. *P < .05; **P < .01; ***P < .001, B: Estimates; CI_95%_: Bias corrected and accelerated confidence interval; *β*: Standardised betas.

In past decades, the TPB (see Figure 1) has demonstrated its usefulness in studies of health-related behaviour [21, 22], including physical activity [23, 24]. However, a limited number of publications reported applications of this theory among children aged between nine and twelve who have not yet entered high school [25–28]. According to these studies, intention, the core construct of the TPB, has been identified as the principal determinant of physical activity among children. Thus, in reference to Ajzen's recommendations for the development of interventions based on the TPB [29], the next step is to investigate the determinants of intention and their related beliefs reflecting the cognitive foundation of the targeted behaviour. To our knowledge, no study in the scientific literature has reported which key beliefs should be targeted in community-based interventions aimed at increasing physical activity among children. Thus, the aim of this study was to identify correlates of regular physical activity as well as key elements to guide the development of such interventions.

### 1.1. Theoretical Framework

This study was based on an extended version of the TPB. According to the TPB, behaviour is predicted by intention and perceived behavioural control when the behaviour is not completely volitional. In turn, intention is predicted by attitude, subjective norm, and perceived behavioural control. Attitude represents the individual's favourable position towards adopting a specific behaviour. Subjective norm is the person's perception of the approval from significant others. Perceived behavioural control is defined as the degree of ease or difficulty with which behaviour can be adopted. Each of these three variables is defined by specific behavioural, normative, and control beliefs. In the present study, only beliefs were assessed, to shorten the questionnaire and because children of this age may not present the cognitive capacities for the abstraction needed to evaluate their behaviour. Also, only beliefs were considered given that they represent potential targets for intervention [30]. In addition to beliefs, other variables known to contribute to the explanation of children's intention or behaviour were also considered (i.e., descriptive norm [31], self-identity [32], and Triandis's concept of facilitating factors [33]). Finally, the influence of direct parental support, parental physical activity level, and body mass index (BMI) as well as children's BMI and past sedentary activities were explored. 

## 2. Methods

### 2.1. Sample and Procedure

A total of 334 fifth graders and 325 parent-respondents were recruited in six schools of an urban region of Quebec, Canada. Participation rates were 84.3% for children and 82.1% for their parents. After the exclusion of some participants (e.g., technical problems with the computer-based questionnaire, absence the day of data collection, misunderstanding of the questionnaire, no corresponding questionnaires of one parent and outliers), the data of 313 children and their participating parents were retained for analysis.

The computer-based questionnaire was completed by the children during school hours. Between 20 to 30 minutes were devoted to this task. Parents completed their questionnaire at home. The questionnaire was completed by one respondent (i.e., father, mother, or a legal respondent). For participation, written consents of both one parent and the child were obtained. This study was approved by the local University Ethics Committee.

### 2.2. Children's Questionnaire

The computer-based questionnaire was developed on an *Asus MyPal A620 PocketPC* (Asus computer international, 44370 Nobel Drive, Fremont, CA 94535, EU) to facilitate comprehension, stimulate participation, and maintain children's attention. This questionnaire was developed following Ajzen's guidelines for developing a TPB questionnaire: the measurement of beliefs based on an elicitation study [34]. Accordingly, semistructured interviews took place with 28 children aged from 10 to 11 on perceived advantages/disadvantages, sources of encouragement, and barriers/facilitators regarding physical activity. Thereafter, a content analysis was conducted by two independent reviewers to classify and identify the children's most important beliefs. For each category of salient beliefs, a frequency of mention was established and those which were the most frequently mentioned (up to 75% of the total) were retained. In a second phase, the questionnaire was evaluated by four experts and twelve children to validate the clarity and comprehension of the items. All psychosocial variables were measured on four-point scales: (1) no, not at all; (2) no, not really; (3) yes, maybe; and (4) yes, for sure and showed moderate to good internal consistency and temporal stability over a two-week reliability test (test-retest). 

Intention was assessed with the following three items: this week…(1) *will you do physical activities? *(2) *will you try to do physical activities?*, and (3) *what are the chances of you doing physical activities?* (*α* = 0.75 and Intraclass coefficient = 0.71). 

Positive behavioural beliefs were assessed by two items: *doing physical activities is…*(1)* fun* and (2) *something to do when I am bored* (Spearman coefficient = 0.24, *P* < .0001). Negative behavioural beliefs were also assessed by two items: *doing physical activities…*(1) is* tiring* and (2) *can cause me bodily injury* (Spearman coefficient = 0.26, *P* < .0001).

Normative beliefs were measured using four items: *do your…* (1) *parents*, (2*) friends*, (3) *other members of your family*, and (4) *teachers encourage you to do physical activities*? (*α* = 0.67 and Intraclass coefficient = 0.70). Descriptive norm was also assessed by four items: *are…*(1) your *friends,* (2*) one of your siblings*, (3) *your father*, and (4) *your mother physically active*? (Intraclass coefficient = 0.63). Self-identity was measured by two items: *do you think you are … *(1) *the sporty type* and (2) *a physically active youth?* (Spearman coefficient = 0.69, *P* < .0001).

In the present study, control beliefs referred to Bandura's concept of self-efficacy [35]. Self-efficacy refers to a child's capacity to overcome perceived barriers to adopt the behaviour. Self-efficacy was assessed by the following four items: *do you think you can do physical activities even if* (1) *they are difficult*?, (2) *you have homework to do*?, (3) *the weather is bad?*, and (4) *you have some others activities to do*? (*α* = 0.66 and Intraclass coefficient = 0.52). Finally, facilitating factors were assessed by mean of eight items: *Is it easier for you to do physical activities if* (1) *you like the physical activities proposed to you*?, (2) *you have equipment at home*?, (3) *your parents enrol you in physical activities*?, (4) *you can do physical activities at school*?, (5) *you have transportation*?, (6) *you are involved in a sports team*?, (7) *your parents encourage you?*, and (8) *you have a friend with you?* (*α* = 0.68 and Intraclass coefficient = 0.48).

Inspired by previous work by Sallis et al. [36], physical activity behaviour was evaluated using a checklist of the most usual sports and physical activities practiced in winter (i.e., the study context). Children had to report if they practiced these activities and how many times they practiced them in the last seven-day period. It was explained to the children that physical activity included any activities or exercise that made them move, breathe hard, and increase their heart rate [37]. Children who reported at least seven periods of physical activity or more were considered active [7, 38]. Finally, children's past sedentary activities were assessed by two items: *yesterday, did you *(1) *watch TV*? (2) *play video/computer games or work on a computer*? Answers were (1) *no*, (2) *yes, for no more than 30 minutes*, (3) *yes, between 30 minutes to one hour*, and (4) *yes, for more than one hour*.

The parents' questionnaire consisted of questions on their regular leisure-time physical activity practices over the last three months [39]. They were also asked to report their weight and height as well as the weight and height of their children. The Body Mass Index (BMI) of parents was estimated using the Canadian guidelines for body weight classification in adults [40] and the BMI of children was estimated using the Cole et al. [41] classification. Finally, parental support for structured activities was measured using the following items: (1) *are you involved in your child's sports organization?*, (2) *are you present when your child is participating in his/her activities?*, and (3) *do you offer transportation to your child?* Scales ranged from no, never (+1) to yes, often (+3).

### 2.3. Statistical Analysis

Hierarchical multiple linear regression analyses were used in the following sequence to predict physical activity: first, the behaviour was regressed on intention and control beliefs (i.e., self-efficacy and facilitating factors); second, descriptive norm and self-identity were added to the model; finally, the influence of other characteristics was tested (i.e., gender, children's and parents' BMI, direct parental support for structured activities, and past sedentary behaviour). To predict intention, the same procedure was applied: first, intention was regressed on the positive and negative behavioural beliefs, normative beliefs, and self-efficacy; second, facilitating factors, descriptive norm, and self-identity were added to the model; finally, the influence of the other characteristics cited above was tested to predict children's intention to be physically active. According to a recent framework proposed by Kremers et al. [42] for the study of energy-balanced related behaviours (i.e., the Environmental Research framework for weight Gain prevention), environmental factors can either be direct predictors of behaviour or mediated by other theoretical constructs. To test this specific hypothesis, a mediation analysis of variables correlated with intention and its determinants identified in previous analyses was performed using a bootstrapping procedure for multiple mediator models [43]. 

Lastly, in order to identify potential targets for community-based interventions, an approach proposed by Von Haeften et al. [30] was adopted. This logistic regression analysis allows identification of the most salient beliefs related to different levels of intention. For this analysis, the intention was dichotomised at the median, allowing the discrimination of beliefs explaining a high level of intention versus a low level of intention. First, intention was regressed on the items of its significant determinants identified in the above multiple regression analysis. This operation was completed separately for each set of beliefs. At the final step, all significant items were added in a final model with the remaining significant items representing the most promising intervention targets.

## 3. Results

### 3.1. Sample Characteristics

The sample consisted of 161 girls and 152 boys with a mean age of 10.4 (*SD* = 0.5) years. The percentages of overweight and obese children were 11.6% and 2.5%, respectively. The mean age of their corresponding parents was 41.9 (*SD* = 4.6) years. The percentages of overweight and obese parents were 32.3% and 7.3%, respectively. In this sample, the mean frequency of physical activity was 15.3 (*SD* = 6.5) periods in the last week, indicating that children participated in two periods of physical activity per day on average. When examining the type of physical activity performed, results indicated that children were engaged primarily in unstructured activities such as playing ball and playing outdoors; only three children (<1%) reported no involvement in these two activities at least once in the last week. Concerning the level of physical activity of the parents, 37.8% of them reported at least three periods of moderate physical activity per week in the last three months.

### 3.2. Correlates of Physical Activity

The correlates of children's physical activity were intention to be physically active and self-identity, explaining 14.9% of the variance of physical activity behaviour. None of the other characteristics were significantly associated with children's behaviour (see Table 1).

### 3.3. Determinants of Intention

The determinants of children's intention to be physically active almost every day of the week were, in order of importance, self-efficacy, self-identity, positive behavioural beliefs, and gender. These four variables explained 47.0% of the variance of intention. The mediation analysis showed that the influence of external variables (i.e., sedentary activity, parental level of physical activity, involvement, presence, and transportation) on children's motivation toward physical activity was mediated by self-efficacy, positive behavioural beliefs, and self-identity, except for parental involvement not mediated by behavioural beliefs (see Figure 1 for the final model). It is noteworthy that the BMIs of children and parents were not correlated with intention and its determinants. Consequently, they were not tested as potential mediating variables.

Because gender had a positive influence on intention, data were reanalyzed separately for boys and girls. The final models for boys and girls were similar, in that self-efficacy, self-identity, and positive behavioural beliefs significantly predicted intention (see Table 2). However, for girls, involvement in sedentary activities was an additional determinant having a negative influence on intention. Nonetheless, the percentage of explained variance was significantly higher for boys than for girls (*z* = 1.97). Consequently, the analysis of key beliefs was performed separately for boys and girls.

### 3.4. Key Beliefs for Community-Based Interventions

For girls, key beliefs related to high levels of intention were as well as: (1) *doing physical activities almost every day is fun *(behavioural beliefs), *I can do physical activities almost each day even if *(self-efficacy), (2) *they are difficult* and (3) *I have homework to do*, and finally (4) *do you think you are a physically active youth? *(self-identity). For boys, there were only two important key beliefs defining motivation: *I can do physical activities almost each day even if *(self-efficacy) (1) *they are difficult* and (2) *I have some other activities to do*.

## 4. Discussion

Results of this study suggest that only children's intention and self-identity explained a small proportion of variance in their participation in physical activities. Nonetheless, this result compared very favourably with previous predictive studies based on the TPB among children [25–28] and give new insight on key beliefs associated with high intention to be physically active. In these previous studies, the explained variance did not reach 10%, except for one study by Rhodes et al. [25], in which 35% and 50% of the variance of behaviour was explained by intention, perceived behavioural control, and past behaviour for the two follow-ups, respectively. None of the parental variables contributed to the prediction of either behaviour or intention. This result is quite surprising, given that many studies have observed the positive influence of multiple dimensions of parental support on children's and adolescents' physical activity behaviour [44–47]. In fact, the results of the mediation analysis suggest that the influence of these variables on children's motivation toward physical activity is mediated by cognitions. Consequently, parental support appears to play a significant role in the development of a high level of self-efficacy, positive attitude, and a perception of being the sporty type or an active youth. Such results confirm previous observations that indicated that environmental factors are mediated by the TPB variables [48, 49]. It is also interesting to note that self-identity towards being the sporty type and an active child plays a significant role in explaining behaviour as well as intention. This suggests that promoting a positive image of being an “active” youth could be an effective way of encouraging participation in regular physical activity.

The importance of intention suggests that educational strategies aimed at increasing children's motivation remain an important strategy to promote physical activity. To increase motivation, however, some additional information from the structure of beliefs is required. Results of the present study suggest that the strategies adopted should be different for girls than for boys. Indeed, a deeper analysis of their key beliefs revealed that although two of the important barriers to physical activity were similar for boys and girls (i.e., perceived difficulty and time management), the cognitive foundation of the motivation toward physical activity was slightly different. Indeed, for girls, having fun while participating in physical activities and perceiving themselves as active individuals were two additional significant elements associated with positive intention, whereas these two aspects were not salient for boys. Consequently, parents and physical educators should make sure that girls have positive experiences with physical activity. It would be important to facilitate access to a variety of activities allowing girls to discover physical activities in line with their personal interests and in which they can excel. The creation of contexts in which children, and girls in particular, have the possibility to explore a set of physical activities and choose their favourite could stimulate more enthusiastic participation. In this study, the activities most often reported for girls were playing outside (99%), dancing (73%), playing ball (72%), and skating (68%). It was also observed that the frequency of sedentary activities was negatively associated with physical activity in girls but not in boys. Decreasing time spent in sedentary behaviour has been proposed as an effective strategy to increase level of physical activity in youths [50, 51]. In the context of the present study, it appears that promoting a decrease in sedentary behaviour would prove effective among girls only, given that the motivation of boys toward physical activity is not influenced by watching TV and playing video/computer games or working on a computer. 

Children who perceived having many other activities or homework to do demonstrated less intention to be physically active. This observation supports the idea that the allocation of priority periods during or after school and on weekends, when children could be physically active, should help to increase their motivation towards physical activity. To minimize difficulties related to the physical activities, it would be determinant that children develop and practice skills during physical education classes. Indeed, physical education is taught to create a positive social environment, especially for girls, and to facilitate skills and confidence for physical activity in children. Hence, the acquisition of such skills could increase children's feelings of competence and, as such, enjoyment. Also, using some of Bandura's strategies aimed at increasing self-efficacy such as increasing gradually the level of difficulty could help children to develop feelings of mastery of physical activities, thereby increasing their perception of self-efficacy [13].

The above suggestions for the promotion of physical activity among children are likely to be effective. Indeed, a recent mass media campaign (the VERB campaign), aimed at promoting physical activity among children aged from 9 to 13 years and developed and tested by the Centers for Disease Control and Prevention (CDC), showed positive results. This mass media intervention, based on TPB and SCT, was designed to encourage playing, promote physical activity as fun, cool, and socially appealing behaviour, and to provide abilities to overcome barriers to physical activity [52]. After one year, their results indicated that children who were aware of the campaign were involved in 34% more free-time physical activity periods than children who were not aware of the campaign [53]. Also, children exposed and aware of the campaign remained more active and had more positive attitudes toward physical activity behaviour after follow-up two years later [54].

Traditionally, environmental variables have not received much attention within the TPB framework. In this study, the implication of parents in their children's sports organization was analyzed. Although none of the parental variables (i.e., involvement, transportation, presence, and physical activity level) and children's related perception (i.e., facilitating factors) of environmental variables were significant correlates of children's physical activity, this study provides some explanations for this lack of direct environmental influence. Indeed, the mediation analysis provided results well aligned with previous observations that environmental variables could be mediated by intention or self-efficacy rather than having a direct effect on behaviour [45, 55]. In this respect, this study has added information regarding the interplay among environmental factors (family-related factors) and cognitions. However, more research is still needed to understand the relationships between environmental factors, psychosocial variables, and children's physical activity behaviour.

Finally, some limitations of this study must be noted. First, although Rhodes and Plotnikoff [56] documented the relevance of proxy measures of physical activity as an expression of future or current physical activity behaviour, a longitudinal study would allow stronger conclusions regarding the direct predictors of children's physical activity or the moderating effects of environmental factors on this behaviour. Secondly, all information was self-reported. Consequently, because children are sensitive to social desirability bias [57], they could have responded more favourably to psychosocial variables and overestimated their physical activity participation. Finally, these findings should not be generalized to the child population, since the study was conducted among a convenient sample recruited in specific schools. 

To conclude, the present study provides promising theory-based information on ways better to promote the regular practice of physical activity among children. In particular, emphasis should be placed in the development of self-identity regarding physical activity and the development of children's motivation by focusing on the management of specific barriers to physical activity such as time management associated with conflicting activities and the perceived difficulty of physical activities. Also, it is important to ensure that girls have positive experiences in physical activity and identify themselves as active young girls.

## Figures and Tables

**Table 1:** Hierarchical regression analyses for the prediction of behaviour.

Variables	Model 1	Model 2	Model 3	Final model
*Behaviour*	Standardised betas (*β*)			
Intention	0.25***	0.20**	0.22**	0.25***
Self-efficacy	0.14**	0.10	—	—
Facilitating factors	0.04	—	—	—
Descriptive norm		0.00	—	—
Self-identity		0.16**	0.16*	0.19**
BMI (children)			0.01	—
Gender			0.02	—
Involvement			0.02	—
Presence			0.01	—
Transportation			0.08	—
Parents' level of PA			−0.00	—

*R* ^2^	0.13	0.14	0.11	0.14

PA: physical activity.

**Table 2:** Final models of the determinants of intention according to gender.

Variables	Boys	Girls
	Standardised betas (*β*)	
Self-efficacy	0.49***	0.28***
Self-identity	0.20**	0.28***
Positive Behavioural beliefs	0.20**	0.18**
Sedentary activities	—	−0.13*

*R* ^2^	0.55	0.38
